# Correction: Family support after a family member’s suicide: A qualitative exploration

**DOI:** 10.1371/journal.pone.0351573

**Published:** 2026-06-10

**Authors:** The PLOS One Staff

The Data Availability statement for this article is incorrect. The correct statement is: The datasets generated and analyzed during the current study are not publicly available due to ethical restrictions aimed at protecting participant confidentiality. In accordance with the terms approved by the Ethics Commission at Ulm University (application number: 374/18), participants consented to the use of pseudonymized interview data for scientific analysis and the publication of selected anonymized quotations in scholarly outputs but did not consent to full public disclosure of the transcripts. A minimal dataset supporting the study’s findings is provided in S2 Table (found in the Supporting information), which contains additional anonymized quotations and full versions of quotations cited in the manuscript. Access to the complete pseudonymized transcripts may be granted to qualified researchers upon reasonable request, subject to review and approval by the responsible institutional bodies of Ulm University, in consultation with the Data Protection Officer. Formal inquiries regarding data access should be directed to the Research Secretariat of the Section Public Mental Health, Department of Psychiatry and Psychotherapy II, Ulm University (email: sekretariat-pmh-psy2@uni-ulm.de), which will coordinate the review process and ensure compliance with applicable data protection regulations.

The publisher apologizes for the errors.
