# Supplementary material for: Family support after a family member’s suicide: A qualitative exploration
Source: PLoS One. 2025 Oct 23;20(10):e0334964. doi: 10.1371/journal.pone.0334964 (PMC12548858; doi:10.1371/journal.pone.0334964)
Supplement: S1 Table — (PDF) [file pone.0334964.s001.pdf]

S1 Table. Overview of interview themes and narrative prompts

Theme	Summary / relevance	Narrative prompt	Aspects explored<br>(via optional follow-up questions)
**1. Starting the conversation: relationship with the deceased**	This section aimed to explore the participant's relationship with the deceased, including its nature, meaning, and significance in the participant's life.	Can you tell me a bit about [name]? What was your relationship like?	Nature and depth of the relationship<br><br>Role of the deceased in the participant's life
**2. Experience of the loss**	This section focused on the participant's experience of the death itself and its emotional impact—both immediate and over time.	Can you tell me—if you're comfortable with this right now—about the time when [name] died? Please, as we discussed earlier, you just go where you want to go and talk about whatever feels right for you.	How and from whom they learned about the death<br><br>Emotional reactions and initial coping<br><br>Early support from others<br><br>Progression of the grief process over time
**3. Close social contacts**	This section explored how family members and close friends responded to the death, and how these interactions were experienced by the participant—both emotionally and relationally.	How were things with the people close to you after [name]'s death? How did you experience those relationships during that time?	Supportive and unsupportive behaviors and experiences<br><br>Changes in family dynamics<br><br>Evolving friendships<br><br>Feelings of connection, distance, or strain

**4. Wider social network**	This section focused on responses from acquaintances, neighbors, colleagues, and other more distant social contacts. It also explored participants' perceptions of these responses, and their emotional or social impact.	How did your wider circle, including acquaintances or more distant contacts, respond?	Initial reactions<br><br>Hurtful or supportive interactions<br><br>Changes within the wider social network<br><br>Impact of these reactions on the participant<br><br>Disclosure of the cause of death
**5. Social support experiences**	This section examined the types of support participants received from others following the loss. It highlighted both helpful and difficult experiences, including moments when participants felt supported, overwhelmed, or left alone.	Who was there for you after the loss—or tried to be? What kinds of support did you experience?	Emotional and practical forms of support<br><br>Key support figures and their roles<br><br>Surprising sources of help or disappointment<br><br>Experiences of feeling left alone, overwhelmed, or isolated
**6. Seeking and accepting support**	This section addressed how participants navigated the process of seeking or accepting help after the loss. It explored internal and external barriers to accessing support, as well as personal attitudes toward vulnerability and openness.	How was it for you to ask for or accept help after the loss—if at all? Were there moments when you needed support and reached out?	Ease or difficulty in accepting support<br><br>Initiating conversations or asking for help<br><br>Personal openness and willingness to be vulnerable

			Social, cultural, or personal influences on help-seeking
**7. Formal support**	This section explored participants' experiences with formal and peer-based support services following the suicide, such as therapy, counseling, or grief groups. It also addressed decisions to seek, delay, or avoid such help, and factors that shaped those decisions.	Did you make use of any kind of professional or organized support after the loss—like therapy, a support group, or something else?	Type of services accessed (e.g., therapy, support groups, counseling)<br><br>Positive or negative experiences with professional or peer support<br><br>Reasons for seeking, delaying, or avoiding support<br><br>Access barriers (logistical, emotional, or structural)
**8. Perceived support needs**	This section invited participants to reflect on what bereaved individuals most need after a loss by suicide, drawing from their own experiences. It also explored how both informal and formal support systems could be improved.	Thinking about your own experience, what kind of support do you feel is most important for someone who has lost a person close to them to suicide?	Helpful experiences and unmet needs<br><br>Wishes or advice for others in similar situations<br><br>Gaps in social and professional support systems<br><br>Suggestions for change or improvement
**9. Additional topics / comments**	This open-ended section allowed participants to share anything they felt was important but had not yet been addressed. It provided space	We're now almost at the end of the interview. Is there anything else you'd like to share—	Unaddressed thoughts or concerns

	for further reflections, insights, or experiences that did not emerge earlier in the conversation.	something that hasn't come up yet, but feels important or relevant to your experience?	Further personal insights on grief and support<br><br>Additional stories or reflections
**10. Closing the conversation**	This final section was used to close the interview with appreciation. Participants were thanked for their openness and time, reminded of available support resources, and encouraged to reach out if anything related to their participation caused distress. They were also asked whether they were interested in receiving study results.	Thank you so much for everything you've shared today. Before we finish, I'd like to briefly remind you of the materials we talked about earlier, including how to access support if anything feels difficult afterward. Would you be interested in hearing about the study results once the project is complete?	Expressing gratitude and closure<br><br>Reminders of available support services<br><br>Invitation to receive results or stay informed about the project
