# Supplementary material for: Family support after a family member’s suicide: A qualitative exploration
Source: PLoS One. 2025 Oct 23;20(10):e0334964. doi: 10.1371/journal.pone.0334964 (PMC12548858; doi:10.1371/journal.pone.0334964)
Supplement: S2 Table — (PDF) [file pone.0334964.s002.pdf]

S2 Table. Coding system for the category of *family support* with full and additional exemplary quotes

Theme	Code	Subcode	Exemplary quotes (full versions and additions)
Contextual factors	Grief reactions and coping patterns	Facade of strength and normalcy	*My mother took over everything. To relieve my father and my grandma. And yes, I was pretty much alone with it. No one could take care of me, my mother was completely absorbed in her tasks, doing everything and showing no weakness. (P01 / female, 38, grandfather)*<br><br>*The fear, as a child back then, was very present in me, that my parents, that they could have become completely consumed by that [their grief]. You often hear that they themselves might become depressed or withdraw from life. That, of course, wouldn't have been much better than how everything quickly and normally continued for the rest of us. (P04 / male, 39, brother)*<br><br>*And my parents, they functioned. My father went back to work relatively quickly. The daily routine didn't really change. My mother kept going. She cooked every day, as she always did. So, in a way, that was relatively normal. (P03 / female, 23, brother)*<br><br>*So, my mother was outwardly cold; I think it was like her protective shield. (P01 / female, 38, grandfather)*
		Avoidance and withdrawal	*The younger one didn't talk about it at all, he didn't say a word. If he noticed us discussing it, he either remained silent or left. (P06 / female, 35, mother)*<br><br>*And my father, you couldn't really talk to my father about it. (P01 / female, 38, grandfather)*
		Disengagement	*But otherwise, my father succumbed to alcohol during that time. He fell into a real hole. And then there was constant trouble at home between my father and mother. They became very estranged during that time. (P02 / female, 37, uncle, godfather, father)*

		And my father and grandma were just complete wrecks, completely out of order. (P01 / female, 38, grandfather)
	Secrecy	*The only one who has repressed it again is my mother, my mother-in-law, my wife's mother, is very devout Muslim, living in [country]. She couldn't accept it at all. It's one of the gravest sins to take one's own life in their belief. And you could tell that she repressed it so much that it was as if she didn't know about it a few weeks later. (P14 / male, 59, son)*<br><br>*For my mother, for example, it's incredibly important. She simply says: 'It wasn't suicide.' That helps her, essentially. (P11 / male, 60, daughter)*
Shifts in family dynamics and relationships	Strengthened bonds	*I believe my brother and I, we became closer. (P05 / female, 33, father)*
	Reduced contact and estrangement	*And then there was the rupture. My father ultimately broke ties with the extended family, because he couldn't stand such foolish talk, he said. There were comments like 'Why didn't he [the deceased] get on a motorcycle and crash into a bridge pillar?', or 'Why didn't he hang himself in the woods?', and all sorts of things like that. And my father couldn't handle that. At some point, I also said, 'No, I just don't want anything to do with these people anymore.'(P02 / female, 37, uncle, godfather, father)*
Support roles and responsibilities	Balanced mutual support	See code *mutual comfort and togetherness.*
	Division of support roles and resources	*My brother, who was on-site, took care of a lot, organized much, because he could quickly go to the apartment [of the deceased] for documents and such. And yes, I think I was more, actually, as it is perhaps obvious, the*

	one who comforted or tried to provide emotional support. Yes. (Interviewer: Did you feel that someone was also taking care of you?) I took care of myself. (P05 / female, 33, father)<br><br>*So, I would say my father mainly provides support. He tends to keep his emotions to himself. And my mother expresses all her emotions outwardly. (P03 / female, 23, brother)*
Uneven support capacities	*I had to function, I had my mother who couldn't cope, I had my child, I had my household. I had to function. (P02 / female, 37, uncle, godfather, father)*
Parenthood and childcare	*I had an experience with the youth welfare office where I asked if there is any support for the children, I would have liked a family helper. Someone who comes to the house regularly and takes care of the children, but not in the sense that they encourage them to talk, but rather someone who plays with them, takes them on outings, helps them with their homework from time to time. So, not a tutoring service-. I asked if there is any support for the children, someone who simply participates in the children's lives and keeps an eye on them together with me, to make sure they are doing well. This responsibility lies solely with me, and I find it really challenging. The youth welfare office told me that such support exists, but only when a child is already in trouble and showing behavioral issues, like falling behind in school, having ADHD problems, showing aggression, even having suicidal thoughts, bulimia, anorexia, or something like that. Only in these cases. Preventively, it doesn't exist, and I didn't get any support. So when it comes to supporting and accompanying the children, I am left on my own. Someone, who also takes some responsibility off my shoulders, it doesn't exist. Where maybe the soul, only the soul is in distress or the soul is threatened. So exactly, I don't have anyone for that, I wouldn't know-. I can't just go to a child psychologist four times a week with four children on a whim. First, I wouldn't get appointments, second, they don't really want to, third, the logistical burden is way too great. And if I couldn't handle it now, I could fall into depression myself, and then the children would be completely on their own. And I want you to please take note of this, urgent action needs to be taken by authorities as well, in my opinion, to call and ask if there is a need, if they can help. So, even a youth welfare office, I think, they should also find out*

about the death of a parent, or I don't know if they find out, but I was even there. So, I brought up the topic, and they are somehow not responsible for the children who have not become psychologically or psychiatrically problematic yet, especially not for children, so-. I would have to pathologize one of my children to possibly get help. And I don't want to subject them to that pressure, because they are doing the best they can. (P08 / female, 45, husband)

So, I notice that my mother has a strong desire to protect me, but she doesn't try to show it. With more or less success. (laughs) But I give my parents credit for at least trying, yes. (P03 / female, 23, brother)

Because sooner or later, the question will arise. And of course, we-, or I, in a way, am a little afraid of it too. What do you tell them then? And what do you say to a six-year-old, or when she asks in one or two years, a seven- or eight-year-old girl? Had ... siblings at one time too. Do you practically tell children what he-, what he died of or what illness he had? Or do you say, 'It was an accident' or something like that? (P04 / male, 39, brother)

I think it occurred to me that this is a topic: How do I deal with my child? So that was difficult. How do I explain to my child what happened? He was nine. So, I think, even today, it's a bit difficult for me to deal with. Because I think I may have done something wrong there. That's still a bit difficult. I would have liked to have some help with that. How do I handle my child when such a situation occurs? But that was-. I was so shocked. I couldn't convey it at all. It was crazy. And I think he sensed it. (4 seconds) And I would have liked it if we could have been more open with each other. But with a nine-year-old child: What do you do right and what do you do wrong? I found that really difficult now. But no one took that away from me either. My husband couldn't deal with him, and neither could I, really. So, I found that quite difficult. What do I tell him about what happened? He never really asked either. He probably knew something was strange. So, I think, in hindsight, that's what I find the most difficult. That I couldn't work through it. Not even for him. (P07 / female, 55, father)

Characteristics	Supportive family experiences	Mutual comfort and togetherness	*I was just totally sad and had to cry, so I withdrew because I didn't want to do it openly in front of the children. And then they discovered me after a few minutes and then-, it was quite astounding. The little one came first and says-, he's very outspoken, and then he called all the others. 'Mommy is crying, mommy is crying.' And he brought me a tissue and then disappeared again. And then the girls came and sat with me, and actually really comforting me. And the oldest gave me a look that said, 'We're all in this together.' That was a really good feeling. (P08 / female, 45, husband)*<br><br>*My husband and my child because they were the two who were ultimately there. (P02 / female, 37, uncle, godfather, father)*<br><br>*The one thing that fascinated me, my daughter said to me, when my wife-. On the first day, she said, she come to sleep at my place. She doesn't want to leave me alone. Okay, I said. [Son in law`s name] said, he is a bit distant, but he said, 'If you're sleeping there now, then I'm sleeping here too.' Then I said, 'Yes, that's okay.' I said, '[Name], if you want, you can sleep here too.' The first four or five nights, both of them slept at my place, even until the funeral, exactly. Slept at my place every day, yes. It was, I have to say, it was nice. (P15 / male, 64, wife)*<br><br>*Yes, we are already very close, and that is what helps us. It was, of course, shocking for them that life can change so quickly. And it helped me that I have daughters and my wife. And I think I help them too, I hope so. (P12 / male, 57, brother)*<br><br>*Our daughter hasn't been talking walks outside with us for years (laughs). So my husband and I have always walked a lot outside. But it was still very nice that she walked with us those first two days. (P09 / female, 48, son)*
		Family conversations	*And yes, we all considered whether we were to blame or someone else. We all went through the books. My son less, but my daughter and my wife. We all pored over her diaries and actually, we got to the point quite quickly*

		where we said, and eventually we said: 'Actually, we should have known, but we didn't.' So it wasn't such a classic blame assignment. It was simply a disease that we didn't recognize. It could have been cancer or something else. (P11 / male, 60, daughter)<br><br>*So I was very much in exchange with my mother. That was actually the most effective, I would say now. (P07 / female, 55, father)*<br><br>*But then, when I was at home, especially when I was with my brother, it was somehow a little bit better. And when we could talk about how the evening actually went for him too. So it was important to me to be able to imagine or get a better picture of the evening, of what exactly happened, how things unfolded. (P05 / female, 33, father)*
	Involvement of extended family	*We cleared out the room with both my brothers on the day, well, the second day, to find some closure. Because at that moment, doing it alone, I wouldn't have been able to manage it. Just entering the room alone was already... yes-. (P13 / male, 60, son)*<br><br>*My mother-in-law loves to cook and is very nurturing. I do take up her offer to cook lunch. It means I can spend time with [son´s name], have a delicious meal, and not have to stretch myself even further as I already do. So, I definitely accept such things. (P02 / female, 37, uncle, godfather, father)*
Strained or insufficient support	Emotional distance and limited communication	*In our family, warmth was simply not present. Let's say, were never allowed in our family. And even to this day, unfortunately, the act or the death of my brother hasn't led my parents to think differently about life or to set different priorities. It just was what it was. So, emotions were never to be expressed or allowed. That's why I wouldn't have felt comfortable in those days, not even to cry or talk about it with my parents. (P04 / male, 39, brother)*

	They were always quite-. Well, we weren't lacking in money, let's put it that way, but we never really got a hug in our lives, so to speak. And it was similar in the way my parents behaved during the grieving process. (P04 / male, 39, brother)<br><br>*So, it means that one morning, I came out of the shower and suddenly my father was in the bathroom, and that's when he saw my self-harm cuts. He didn't say anything to me; I think he just couldn't handle it. Of course, now I understand that it's not easy. It was briefly like, 'What is this, why?' I'm not even sure if he asked why, but rather just said, 'Don't do such things.' And that was pretty much it. But there was no suggestion of psychological help, or even just some advice, nothing at all. Looking back, I think there was fear because my mother was so heavily dependent on alcohol. Now I know that my father was also an alcoholic, but it manifested differently in him. They were probably afraid that it might come out, and in the end-. You know what can happen, children can be taken away, or whatever. I believe they were also afraid of it becoming public. And that definitely played a role, along with their own unwillingness to deal with their issues. (P01 / female, 38, grandfather)*<br><br>*And he wasn't able to talk to me then either, I think. I believe I didn't speak to him for a while. For me-. I think it was somewhat silent in those days following the news and also the days after that. I actually, didn't want him around. I didn't want him to participate in anything at all. I would have preferred him not even being at the funeral. It was almost repulsive to me. That's harsh, but I just didn't want him sitting next to me. It was quite crazy. (P07 / female, 55, father)*
Pre-existing family strain	*So in my family, everything that was ignored simply didn't exist. I have to say, my mom was severely addicted to alcohol, even before, and what happened with my grandpa just added to it; that's when it really started. But the accusations that it was like that, she always directed at me-. Even before, but afterwards, she really told me, like directly. (Interviewer: So, are you saying that you were blamed or held responsible?) Exactly, I was supposed to have been the trigger. And for me, it felt like-. Now I'm to blame for what my grandpa did. I'm to*

	blame for what my grandpa did to my mother, how it affected her, making it worse. It was like a spiral of guilt. And my self-harm was mentioned once: 'Don't do that anymore, it's not good.' But there was no suggestion of psychological help, or even just some advice, nothing at all. (P01 / female, 38, grandfather)<br><br>*Now, of course, I understand that they were worried, but back then I couldn't perceive it. I honestly felt more like a burden. Like, now I'm also a burden here. I've always been-. My father always told me I was a persistent person. I don't know, I just felt totally annoying, always felt annoying. And probably-. I mean, sure, they had to process it themselves, and then their daughter comes along and makes a mess of things. (P01 / female, 38, grandfather)*
Marginalization	*I'm not sure. Something else came to mind, I don't know if it matters, but sometimes I feel that in my extended family, which isn't very big, but includes aunts, grandma, and so on, that-. I'm still seen as the child in the family. And I'm not asked how I'm doing. My mother is asked how I am, and my father is asked how I am, but not me. I'm still treated like the child who doesn't understand. Or who doesn't have to handle things. Or can't handle things. Or whatever. So that means-. Conversely, nothing is expected of me. Which can be a pleasant thing. But also means I'm not trusted to do things. And to some extent, maybe even overlooked. (P03 / female, 23, brother)*
Discrepancy between expected and received support	*My relationship with my husband just didn't work out. We had some issues-. Ultimately, for various reasons, including this one, we eventually separated. I would say that certainly one reason was that he couldn't support me emotionally at all. It was very difficult, he-. I remember sitting on the phone waiting for news about my brother: 'What's going on? What's happening now?' And my husband just went to bed. (laughs) And then I thought, 'Uh, something is definitely not right here.' But of course, I didn't want to-. I was so disappointed that he didn't go through it with me, I thought, 'Wow, now it's really over.' It was such a shock or a-. It was also a terrible experience, I must say. I was really affected. (P07 / female, 55, father)*

		And then I also noticed that a bit later, a few days later, as the news started to spread, of course, you start to hear about it. But then suddenly-. I have a lot of contacts. Family-wise. We have a very large family. I have many cousins. But during that time, I realized that there were only a few contacts, maybe two, who reached out to me. It was as if suddenly no one was writing to me anymore. (P06 / female, 35, mother)
	Desire for proactive support	*Which is what I would have expected from many others, from my siblings or from old friends. You don't always have to talk about suicide. If I don't want to visit someone, or if I don't want to be visited, they could still say, they could still say, 'Come on, let's go for a walk for an hour, or meet somewhere, anywhere, and just walk and talk a bit.' (P15 / male, 64, wife)*<br><br>*But otherwise, in the family, yes. I'm also the godmother of my cousin's daughter and so on. So, there is a good relationship. But of course, I would have wished for a message from them, maybe a bit sooner. And I was quite disappointed that nothing came at all. (P06 / female, 35, mother)*
