# Supplementary material for: Family support after a family member’s suicide: A qualitative exploration
Source: PLoS One. 2025 Oct 23;20(10):e0334964. doi: 10.1371/journal.pone.0334964 (PMC12548858; doi:10.1371/journal.pone.0334964)
Supplement: Attachment — Submitted filename: Response to Reviewers.pdf [file pone.0334964.s004.pdf]

Response to Reviewers

PLOS ONE
Manuscript ID: PONE-D-25-24683
Title: *Family support after a family member's suicide: A qualitative exploration*
Authors: Franziska Marek, Nathalie Oexle

We sincerely thank the editor and reviewers for their thoughtful and constructive feedback on our manuscript. Below, we respond to each comment in turn. Editor and reviewer comments are shown in **bold**, followed by our response and excerpts from the revised manuscript (edits highlighted in yellow) with line numbers.

Journal Requirements

Journal Requirement 1

When submitting your revision, we need you to address these additional requirements.
1. Please ensure that your manuscript meets PLOS ONE's style requirements, including those for file naming. The PLOS ONE style templates can be found at https://journals.plos.org/plosone/s/file?id=wjVg/PLOSOne_formatting_sample_main_body.pdf and https://journals.plos.org/plosone/s/file?id=ba62/PLOSOne_formatting_sample_title_authors_affiliations.pdf

Author response:
We confirm that the manuscript has been revised in accordance with PLOS ONE's formatting and file naming requirements, as outlined in the provided templates.

Journal Requirement 2

In the online submission form, you indicated that [requests may be directed to the corresponding author and will be reviewed in accordance with institutional and ethical guidelines.].
All PLOS journals now require all data underlying the findings described in their manuscript to be freely available to other researchers, either 1. In a public repository, 2. Within the manuscript itself, or 3. Uploaded as supplementary information.
This policy applies to all data except where public deposition would breach compliance with the protocol approved by your research ethics board. If your data cannot be made publicly available for ethical or legal reasons (e.g., public availability would compromise patient privacy), please explain your reasons on resubmission and your exemption request will be escalated for approval.

Author response:
Our study is based on qualitative data from in-depth, pseudonymized interview transcripts. Although the transcripts have been carefully pseudonymized, they contain contextual details that, in combination, could compromise participant confidentiality if shared in full. According to the terms approved by our institutional ethics committee (Ethics Commission at Ulm University), participants consented to the use of pseudonymized interview data for scientific analysis and to the publication of selected, anonymized quotes in scholarly outputs, but did not consent to the full transcripts being made publicly available. In light of these ethical restrictions, we are unable to deposit the complete datasets in a public repository. We have instead provided detailed supporting information (S2 Table) containing additional anonymized quotes and full versions of

quotes cited in the manuscript, to enhance transparency and rigor. In addition to these ethical restrictions, the interview transcripts are in German, which further limits the feasibility of public data sharing.
In response to the journal's data policy requirements, we have revised the *Data availability statement* in the manuscript (lines 953–968) to explicitly meet all PLOS ONE criteria. The updated statement specifies:
1. The reason for the restriction (protection of participant confidentiality),
2. The name of the responsible institution (Ulm University), and
3. A non-author, institutional contact email for data access requests, in addition to the corresponding author as a secondary contact.

Revised manuscript excerpt (Data availability statement, lines 953–968):
The datasets generated and analyzed during the current study are not publicly available due to ethical restrictions aimed at protecting participant confidentiality. Although all interview transcripts have been pseudonymized, they contain contextual details that, in combination, could potentially allow for participant identification. In accordance with the terms approved by the Ethics Commission at Ulm University (application number: 374/18), participants consented to the use of pseudonymized interview data for scientific analysis and the publication of selected, anonymized quotations in scholarly outputs, but did not consent to full public disclosure of the transcripts. A minimal dataset supporting the study's findings is provided in S2 Table (found in the supporting information), which contains additional anonymized quotations and full versions of quotations cited in the manuscript. Access to the complete pseudonymized transcripts may be considered for qualified researchers upon reasonable request. Formal inquiries regarding data access and restrictions may be directed to the Research Secretariat of the Section Public Mental Health, Department of Psychiatry and Psychotherapy II, Ulm University (E-mail: sekretariat-psyII@uni-ulm.de). The corresponding author may be contacted as a secondary point of information.

Journal Requirement 3
Please review your reference list to ensure that it is complete and correct. If you have cited papers that have been retracted, please include the rationale for doing so in the manuscript text, or remove these references and replace them with relevant current references. Any changes to the reference list should be mentioned in the rebuttal letter that accompanies your revised manuscript. If you need to cite a retracted article, indicate the article's retracted status in the References list and also include a citation and full reference for the retraction notice.

Author response:
We have reviewed and revised our reference list and added several references to the revised manuscript (e.g., references 6 and 93–97) in response to reviewer comments. These additions are described in detail below.

Reviewer's Responses to Questions

1. Is the manuscript technically sound, and do the data support the conclusions?
The manuscript must describe a technically sound piece of scientific research with data that supports the conclusions. Experiments must have been conducted rigorously, with appropriate controls, replication, and sample sizes. The conclusions must be drawn appropriately based on the data presented.

Reviewer #1: Yes

Reviewer #2: Yes

Author response:
/

2. Has the statistical analysis been performed appropriately and rigorously?

Reviewer #1: Yes
Reviewer #2: N/A

Author response:
/

3. Have the authors made all data underlying the findings in their manuscript fully available?
The PLOS Data policy requires authors to make all data underlying the findings described in their manuscript fully available without restriction, with rare exception (please refer to the Data Availability Statement in the manuscript PDF file). The data should be provided as part of the manuscript or its supporting information, or deposited to a public repository. For example, in addition to summary statistics, the data points behind means, medians and variance measures should be available. If there are restrictions on publicly sharing data—e.g. participant privacy or use of data from a third party—those must be specified.

Reviewer #1: No
Reviewer #2: Yes

Author response:
Our study is based on qualitative data from in-depth, pseudonymized interview transcripts. Although the transcripts have been carefully pseudonymized, they contain contextual details that, in combination, could compromise participant confidentiality if shared in full. According to the terms approved by our institutional ethics committee (Ethics Commission at Ulm University), participants consented to the use of pseudonymized interview data for scientific analysis and to the publication of selected, anonymized quotes in scholarly outputs, but did not consent to the full transcripts being made publicly available. In light of these ethical restrictions, we are unable to deposit the complete datasets in a public repository. We have instead provided detailed supporting information (S2 Table) containing additional anonymized quotes and full versions of quotes cited in the manuscript, to enhance transparency and rigor. In addition to these ethical restrictions, the interview transcripts are in German, which further limits the feasibility of public data sharing.
In response to the journal's data policy requirements, we have revised the *Data availability statement* in the manuscript (lines 953–968) to explicitly meet all PLOS ONE criteria. The updated statement specifies:
1. The reason for the restriction (protection of participant confidentiality),
2. The name of the responsible institution (Ulm University), and
3. A non-author, institutional contact email for data access requests, in addition to the corresponding author as a secondary contact.

Revised manuscript excerpt (Data availability statement, lines 953–968):

The datasets generated and analyzed during the current study are not publicly available due to ethical restrictions aimed at protecting participant confidentiality. Although all interview transcripts have been pseudonymized, they contain contextual details that, in combination, could potentially allow for participant identification. In accordance with the terms approved by the Ethics Commission at Ulm University (application number: 374/18), participants consented to the use of pseudonymized interview data for scientific analysis and the publication of selected, anonymized quotations in scholarly outputs, but did not consent to full public disclosure of the transcripts. A minimal dataset supporting the study's findings is provided in S2 Table (found in the supporting information), which contains additional anonymized quotations and full versions of quotations cited in the manuscript. Access to the complete pseudonymized transcripts may be considered for qualified researchers upon reasonable request. Formal inquiries regarding data access and restrictions may be directed to the Research Secretariat of the Section Public Mental Health, Department of Psychiatry and Psychotherapy II, Ulm University (E-mail: sekretariat-psyII@uni-ulm.de). The corresponding author may be contacted as a secondary point of information.

4. Is the manuscript presented in an intelligible fashion and written in standard English?
PLOS ONE does not copyedit accepted manuscripts, so the language in submitted articles must be clear, correct, and unambiguous. Any typographical or grammatical errors should be corrected at revision, so please note any specific errors here.

Reviewer #1: Yes
Reviewer #2: Yes

Author response:
/

Reviewer#1 Comments

This qualitative analysis focuses on the nature of family support available after a relative's suicide, noting the particular issues for adolescents lacking appropriate support within the family, and warning that it cannot be assumed that some people will have any family support available. This was very well written and methodologically generally very sound, although I do have some specific comments below.

Author response:
We thank Reviewer#1 for their careful reading and thoughtful comments. We address each point below.

Comment 1: Abstract
The two overarching themes identified in the abstract do not list any sub-themes in the abstract, so the text that follows this in the abstract does not align itself clearly to each of them: (1) Contextual Factors of Family Support, and (2) Characteristics of Family Support. Editing this to be clear which were sub-themes of which theme would convey the results better.

Author response:
We revised the *Abstract* to explicitly link each overarching theme to its corresponding sub-themes, thereby clarifying the structure and improving alignment with the *Results* section of the manuscript. Specifically, the theme 'Contextual factors of family support' now includes grief reactions and coping patterns, shifts in family dynamics, and the

distribution of support roles. The theme 'Characteristics of family support' now encompasses both supportive experiences—such as emotional closeness, open communication, and shared sense-making—and insufficient support, including the marginalization of grief and emotional neglect. These revisions aim to clarify the thematic structure. To comply with the journal's 300-word abstract limit, we made several minor wording adjustments to streamline the text without altering its meaning or tone. Please see lines 25–35 of the revised manuscript for these changes.

Revised manuscript excerpt (Abstract, lines 25–35):
Two overarching themes were identified: (1) *Contextual factors of family support*, including grief reactions and coping patterns, shifts in family dynamics, and the distribution of support roles; and (2) *Characteristics of family support*, encompassing both supportive experiences—such as emotional closeness, open communication, and shared sense-making—and insufficient support—such as marginalization of grief and emotional neglect, often linked to pre-existing family strain. The availability and quality of support were influenced by protective buffering, relational withdrawal, discomfort surrounding suicide disclosure, and the reconfiguration of relationships after the loss. Extended family members played a significant role in assisting with childcare and relieving emotional burdens on grieving parents, although their support varied depending on attitudes toward suicide and levels of proactive engagement.

<u>Comment 2: Introduction</u>
Important to distinguish between 'exposure' and 'bereavement' when noting "from those who know the deceased to those who experience bereavement".

Author response:
We revised the respective section of the *Introduction* (lines 48–51) to more clearly distinguish between exposure and bereavement, drawing on Cerel et al.'s conceptualization of the *Continuum of Survivorship*. The revised text now explicitly differentiates individuals who knew the deceased, those who are emotionally affected, and those who are bereaved following the loss of someone with whom they had a close attachment. This clarification strengthens the conceptual accuracy of our framing and reinforces the focus of our study on suicide-bereaved individuals. We also added a citation of Cerel et al.'s 2014 publication (line 48), which introduced the *Continuum of Survivorship*, to ensure appropriate attribution and conceptual clarity.

Revised manuscript excerpt (Introduction, lines 48–51):
As Cerel and colleagues [6] have emphasized, suicide loss occurs along a continuum of exposure and impact—from individuals who knew the deceased, to those who are emotionally affected, to those who are bereaved following the loss of a close attachment and experience profound psychological and relational consequences.

<u>Comment 3: Introduction</u>
When considering the perspectives of those from different disciplines (in the introduction and discussion), it is valuable to label the discipline of named authors/papers eg "Building on this perspective, the [identify discipline they belong to] Hayslip and Page….." to be clear about how different disciplinary contributions come together.

Author response:
While the cited scholars primarily work within the fields of psychology, social psychology, or public health—and therefore do not represent entirely separate academic

disciplines in a strict sense—they draw on distinct theoretical traditions that inform the development of our study. To clarify these distinctions, we revised the *Grief as a relational process* section to explicitly describe the conceptual orientation of each contribution: Gilbert adopts a social constructionist perspective on grief; Hayslip and Page draw from family psychology and systems theory to propose a family systems framework; Stroebe and Schut bridge individual and relational models in their *Dual Process Model* of coping with bereavement; and Breen and colleagues advance a transdisciplinary approach that integrates psychosocial bereavement research and public health. These clarifications (lines 68–70; 75–78; 84–86; 93–97) highlight the complementary nature of these frameworks and the conceptual richness they bring to the study of family grief and support.

Revised manuscript excerpt (Introduction / Grief as a relational process, lines 68–70):
Gilbert [13], adopting a social constructionist perspective on grief, conceptualizes the family as a central "arena" of bereavement.

Revised manuscript excerpt (Introduction / Grief as a relational process, lines 75–78):
Building on this perspective, Hayslip and Page [14], drawing from family psychology and systems theory, adopt a family systems framework that emphasizes the concept of *multileveled embeddedness*—the idea that grief unfolds within and across interrelated systems, including the family unit, broader social networks, and cultural or institutional contexts.

Revised manuscript excerpt (Introduction / Grief as a relational process, lines 84–86):
This relational lens is echoed in the revised *Dual Process Model* of coping with bereavement *(DPM-R),* proposed by Stroebe and Schut [15], which bridges individual-level and relational models of coping.

Revised manuscript excerpt (Introduction / Grief as a relational process, lines 93–97):
This shift toward relational understandings of bereavement is further advanced by Breen and colleagues [12], whose work is situated at the intersection of psychosocial bereavement research and public health, reflecting a transdisciplinary approach to grief in the family context. They argue that grief is "a family affair," offering a research- and practice-oriented critique of individualistic paradigms in bereavement theory and research.

Comment 4: Materials and methods
What was the compensation for participants?

Author response:
This information is included in the *Data collection* section of the manuscript (lines 279–280), where it is noted that all participants received €30 as compensation for their participation.

Comment 5: Materials and methods
For the purposive sampling, the authors aimed for a sample size of 20 participants, "which was deemed methodologically appropriate and practically feasible, based on prior

experience with qualitative research on emotionally complex and deeply personal topics". Criteria for purposive sampling were gender, age, relationship to the deceased, and use of formal support services. It sounds as though individuals were chosen on this basis from available volunteers and the rest were registered for the broader DE-LOSS project. However, if 5 were excluded, why not increase from the 15 eligible subjects to a sample of 20?

Author response:
The qualitative sub-study of the *DE-LOSS* project was designed as an exploratory investigation into social support following suicide bereavement, using a purposive sample of 20 participants selected based on characteristics such as gender, age, relationship to the deceased, and use of formal support services. During data analysis, it became evident that five interviews did not align with the specific focus of this manuscript on intra-family support dynamics—a theme that emerged inductively from the data rather than being predefined. Specifically, three participants had experienced the suicide of a partner or close friend outside a shared family context, while two cases involved assisted suicide, which introduced distinct contextual considerations. These interviews were not discarded but instead informed other *DE-LOSS* publications. As the thematic narrowing occurred after data collection had concluded, no additional interviews were conducted to replace the excluded cases. The final analytic sample of 15 participants remains methodologically robust and consistent with prior qualitative studies on suicide bereavement. To clarify this for readers, we added explanatory text to the *Recruitment and sample selection* section (lines 239–245).

Revised manuscript excerpt (Materials and methods / Recruitment and sample selection, lines 239–245):
The excluded interviews informed related publications within the DE-LOSS project, addressing non-familial social support [29] and the experiences of individuals involved in assisted suicide [46]. These exclusions were made during data analysis, as the specific thematic focus on intra-family support emerged inductively rather than being predetermined. Accordingly, the final analytic sample of 15 participants represents a thematically relevant subsample of the purposively recruited group. No additional interviews were conducted, because data collection for the qualitative sub-study had already concluded.

Comment 6: Materials and methods
Were any individuals from 'made families' / chosen families / a family of choice? ie those identifying as LGBTQ who had been alienated by their birth families and had instead built up a network of individuals functioning as a de facto family.

Author response:
In our study, we did not impose a fixed or normative definition of 'family', allowing participants to define 'family' in their own terms—consistent with Breen et al.'s conceptualization, as outlined in the *Introduction* (lines 184–193). The bereavement experiences explored in this manuscript were situated within families of origin or legally recognized kinship structures. To clarify this for readers, we added an explicit note in the *Sample* section (lines 251–252). Additionally, we now address this issue in the *Conclusion*, where we emphasize the conceptual and methodological importance of including chosen families and non-traditional kinship structures in future research (lines 932–942).

Revised manuscript excerpt (Materials and methods / Sample, lines 251–252):
All participants described the bereavement context analyzed here as embedded within a family of origin and/or legally recognized kinship system.

Revised manuscript excerpt (Conclusion on directions for research and practice, lines 932–942):
In this study, all participants described the familial context of their bereavement within a family of origin and/or legally recognized kinship systems. As such, the sample did not include individuals who identified a 'chosen family' [93] as the context of their bereavement. Nonetheless, such experiences are highly relevant—particularly among LGBTQ+ communities, where estrangement from families of origin and the formation of chosen families are well-documented practices and coping strategies in the face of stigma and marginalization [93–97]. These kinship structures may play a vital role in emotional support and grief processes for many individuals. Future research should adopt inclusive sampling strategies and conceptual frameworks to better capture bereavement experiences beyond traditional kinship ties, and to understand how chosen family networks can be strengthened to mitigate isolation and disenfranchised grief.

Comment 7: Materials and methods
NO performed random checks – how was the randomness operationalised?

Author response:
To ensure coding consistency and analytic rigor, NO conducted structured reliability checks across the entire coding system. Specifically, she reviewed multiple coded segments within each main category and code. The number of segments reviewed varied based on the volume of data associated with each code, ensuring that both frequently and infrequently used codes were adequately assessed. We have clarified this process in the *Data analysis* section (lines 303–306) to explain how these checks were operationalized.

Revised manuscript excerpt (Materials and methods / Data analysis, lines 303–306):
To ensure consistency, NO conducted structured reliability checks by reviewing multiple segments within each code. The number of segments reviewed varied according to the volume of material assigned to each code, ensuring representation across both high-frequency and low-frequency codes.

Comment 8: Materials and methods
There was no mention of reflexivity.

Author response:
Reflexivity was an integral part of our qualitative research process. FM maintained reflexive memos throughout data collection and analysis to critically examine assumptions, preconceptions, and the influence of the researcher's positionality. These reflections were discussed within the research team and with the participatory advisory board to promote analytic transparency and reduce the risk of interpretive bias. While reflexivity was previously mentioned in the *Data analysis* section, we have now expanded this description to clarify how reflexive practices were integrated into the analytic process (lines 293–297).

Revised manuscript excerpt (Materials and methods / Data analysis, lines 293–297):

FM documented reflexive memos throughout the study to examine assumptions, preconceptions, and the influence of the interviewer's position. These reflections were discussed within the research team and the participatory advisory board to enhance analytic transparency and strengthen the interpersonal validity of interpretations.

Comment 9: Materials and methods

This manuscript focuses on the category of Family Support, comprising 199 coded segments, but what happened to the other categories? Otherwise it sounds a bit like salami-slicing instead of presenting a coherent whole.

Author response:

The qualitative sub-study was designed as an exploratory investigation into social support following suicide bereavement, using an inductive approach to identify thematically rich domains. As outlined in the manuscript and *Supporting information S1 Table*, the interview guide covered a range of support contexts, including both familial and non-familial relationships. During analysis, the category 'Family Support' emerged as a conceptually rich and distinct area of inquiry, warranting focused, in-depth treatment. Other categories related to non-familial support were analyzed separately and are reported in a related publication (as noted in lines 239–241). This decision reflects the data-driven nature of our analytic process rather than any pre-planned partitioning of results. To clarify this for readers, we have now added an explicit statement in the *Data analysis* section that this manuscript presents *one* category from the broader coding system (lines 322–326). We believe this thematically focused approach enhances conceptual clarity, deepens the study's theoretical contribution, and allows for more targeted practical implications.

Revised manuscript excerpt (Materials and methods / Data analysis, lines 322–326):

This manuscript presents findings from one category of the coding system—*family support*—which comprises 199 coded segments and emerged as a conceptually rich domain warranting in-depth analysis. Within this category, five codes were identified and further refined into 18 subcodes. These codes were grouped into two overarching themes that served to organize the material conceptually (see Table 2).

Comment 10: Materials and methods

Did you capture religion? This was relevant to some quotes/themes so would be relevant to have measured, or to add to limitations.

Author response:

While we included an item on religious belief in the demographic questionnaire, religion and spirituality were not core analytic variables and did not emerge as prominent or recurring themes in participants' narratives. One participant referenced the belief system of their Muslim mother-in-law in the context of family tensions around suicide disclosure (lines 399–401), but religious or spiritual frameworks were not broadly evident across the dataset. However, we agree that religious and spiritual belief systems may play a meaningful role in grief processes and family support. To reflect this potential relevance, we have added a note in the *Conclusion* (lines 943–945), encouraging future research to examine how religion and spirituality may influence support-seeking, meaning-making, and family interactions in suicide bereavement. For transparency, we also clarified in the *Data collection* section that a short demographic questionnaire was administered and included an overview of the variables assessed, including religious belief (lines 261–263).

Revised manuscript excerpt (Materials and methods / Data collection, lines 261–263):
All participants completed a brief demographic questionnaire assessing age, gender, religious belief, migration background, education, employment, living situation, and suicide loss characteristics.

Revised manuscript excerpt (Conclusion on directions for research and practice, lines 943–945):
Religion and spirituality did not emerge as prominent themes in the interviews of this study. Future research could explore how faith, religious identity, or spiritual belief systems influence family interactions, meaning-making, and support-seeking in suicide bereavement.

Comment 11: Results
I find the labels (P01) etc not very helpful in getting a quick sense of age, gender, kinship etc for each quote.

Author response:
To improve the contextual clarity of participant quotes, we have revised the citation format throughout the *Results* section. Each quote now includes the participant's assigned code, gender, age at the time of interview, and the deceased's relationship to the participant (e.g., P01 / female, 38, grandfather). We also updated the corresponding explanation in the *Data analysis* section to reflect this change (lines 327–329).

Revised manuscript excerpt (Materials and methods / Data analysis, see lines 327–329):
All participant quotes are identified by their assigned code, followed by gender, age at the time of interview, and the deceased's relationship to the participant (e.g., P01 / female, 38, grandfather).

Comment 12: Results
Formatting made it hard to know what level of theme/sub-theme one was at.

Author response:
We agree that clarity in presenting thematic structures is essential. Our manuscript adheres to *PLOS ONE*'s formatting guidelines, which allow a maximum of three heading levels. Within these constraints, we structured and formatted the *Results* section as follows:

Level 1: Results (main section; bold type, 18pt font.)

Level 2: Themes (e.g., Contextual factors of family support, Characteristics of family support; bold type, 16 pt font.)

Level 3: Codes (used as subheadings within each theme; bold type, 14pt font.)

To indicate a fourth level—subcodes—we marked these at the beginning of the relevant paragraphs using bolded text followed by a period (e.g., **Facade of strength and normalcy.**). This approach was intended to visually signal subthemes without violating journal sectioning rules. We are, of course, open to alternative formatting suggestions and would be happy to revise the structure if the editors prefer a different approach.

Comment 13: Discussion

Need to mention that some nuances may have been lost in the translation to German, and whether fluency in English of NO/FM mitigated this.

Author response:
As the interview data were originally collected in German and translated into English for publication, we acknowledge that some linguistic nuances—particularly idiomatic expressions and regionally specific language—may not translate directly or fully. Although the research team is fluent in both German and English and took care to preserve intended meaning, subtle connotations may have been lost in translation. We have added a note in the *Limitations* section (lines 794–797) to acknowledge this boundary and increase transparency regarding the cross-linguistic nature of the study.

Revised manuscript excerpt (Discussion / Limitations, lines 794–797):
Finally, as interviews were conducted in German and translated into English for publication, some linguistic nuances may have been lost, despite the research team's bilingual fluency. This limitation applies particularly to idiomatic and regionally specific language, which may not translate directly or fully.

Comment 14: Discussion
Cultural context seems important so when mentioning the findings of other authors eg Creuzé et al. [35] – it would be important to mention where these studies were set.

Author response:
While the referenced studies in the *Discussion* section do not explicitly apply culturally sensitive analytic frameworks—and our own interpretation does not foreground cultural variation—we agree that geographic context remains relevant, particularly for readers and future research interested in cultural dynamics in suicide bereavement and support. To help situate the referenced studies within their geographic and cultural contexts, we have added the country of origin for selected empirical studies throughout the *Discussion* section (lines 642–648, 667–669, 672–675, 685–687, 721–723). These identifiers were applied selectively, focusing on studies where the setting might implicitly shape the experiences or interpretations discussed. We hope these additions enhance transparency and help readers better evaluate the scope and transferability of the findings.

Revised manuscript excerpt (Discussion / Facilitators of supportive family processes, lines 642–648):
These findings align with studies conducted in France by Creuzé et al. [36] and in the United Kingdom by Pitman et al. [38], which highlight how "the family itself" [36] can serve as both an active and latent source of support—the latter providing reassurance that support remains available even when not actively sought. Building on Levi-Belz's [49] work in Israel on perceived belonging and post-traumatic growth, our findings suggest that family cohesion fosters inclusive environments that facilitate equitable access to emotional support within families.

Revised manuscript excerpt (Discussion / Facilitators of supportive family processes, lines 667–669):
Castelli-Dransart [54], in a Swiss study, found that SLS who reject reductive or moralizing interpretations of suicide often come to understand their loss in ways that support personal growth and reengagement with life.

Revised manuscript excerpt (Discussion / Facilitators of supportive family processes, lines 672–675):
Supporting this, U.S.-based studies by Currier et al. [55] and Rozalski et al. [56] identify sense-making as a key protective factor in bereavement after violent deaths, including suicide, mediating the impact of such losses on complicated grief symptomatology.

Revised manuscript excerpt (Discussion / Barriers and gaps in family support, lines 685–687):
Similarly, Breen and O'Connor [57], in an Australian study, found that disparities in emotional expression and memory-sharing may contribute to familial tension and estrangement following sudden loss.

Revised manuscript excerpt (Discussion / Barriers and gaps in family support, lines 721–723):
Similarly, Azorina et al., in a UK-based study, describe how suicide can become "the elephant in the room" [67]—an unspoken subject that nonetheless shapes family interactions.

Comment 15: Discussion
Would be good to add strengths ahead of the limitations. The latter needs to use the term non-representative to be specific, and acknowledge that only 15 of the target of 20 were included, as well as other issues mentioned above.

Author response:
We chose to integrate the strengths of our study throughout the *Discussion* section to highlight them in direct relation to our interpretations and the literature. To avoid redundancy and maintain narrative coherence, we opted against a separate *Strengths* subsection. Several key strengths are explicitly referenced in context:

- In the section *Facilitators of supportive family processes* (lines 639–675), we show how emotional expression and shared sense-making contribute to relational closeness and stigma resilience in bereaved families, thereby extending existing literature on social support in suicide bereavement.
- The section *The role of extended family* (lines 730–752) introduces the novel concept of a *proxy support effect*, highlighting how indirect support—such as caregiving provided to children—can alleviate emotional burden and facilitate grief processing in bereaved parents.
- In the section *Toward an integrative framework for family support in suicide bereavement* (lines 800–873), we highlight our conceptual contribution to grief theory by demonstrating that family support is not a static resource but a relational, co-constructed process shaped by interpersonal and systemic dynamics. We argue for its integration into existing grief models to better reflect the interdependent nature of grief and support within bereaved families.

We now explicitly acknowledge in the *Limitations* section that the sample was non-representative and not intended to support statistical generalization, in line with the aims and epistemology of qualitative research (lines 755–758). In response to the reviewer's comment regarding sample inclusion, we added a clarifying statement in the *Limitations* section noting that although 20 interviews were originally conducted, five were excluded post hoc based on the inductively developed focus on intra-family support (lines 758–762). We agree that this clarification increases transparency regarding sample composition. We also addressed other methodological concerns raised—specifically the

cross-language nature of data collection and translation—and have incorporated these reflections into the *Limitations* section (lines 794–797), as noted previously.

Revised manuscript excerpt (Discussion / Limitations, lines 755–762):
As is typical in qualitative research, the sample was non-representative and not designed to support statistical generalization. Rather, the aim was to explore diverse, in-depth perspectives on family support in the context of suicide bereavement. Although 20 interviews were originally conducted, five were excluded from the present analysis based on an inductively developed focus on intra-family support. While the final sample of 15 participants aligns with qualitative standards and provides a broad range of insights, this post hoc narrowing may have reduced the diversity of perspectives represented in this particular analysis.

Reviewer#2 Comments

Comment 16: Introduction

Thank you for the opportunity to read this insightful and well-executed article on a qualitative research project. I have little to contribute because I find the article to be well-composed, providing a robust background for the knowledge gap, demonstrating a strong familiarity with existing research, and presenting results that offer a significant contribution to work research in a family context. Additionally, the discussion presents implications for both practice and further research. The only minor comment I have is in the Introduction (Lines 63-64), where I believe a reference is needed for the following sentence: "Grief is not only an individual reaction to loss but a relational process embedded within social…"

Author response:
In response to the suggestion, we have added a reference to Breen et al. (2019) in the *Introduction* to substantiate the relational conceptualization of grief within family systems (now, lines 66–67).

Revised manuscript excerpt (Introduction / Grief as a relational process, lines 66–67):
Grief is not only an individual reaction to loss but a relational process embedded within social systems—most notably, the family [12].
